# Homologous recombination is involved in the diversity of replacement flexible genomic islands in aquatic prokaryotes

**DOI:** 10.3389/fgene.2014.00147

**Published:** 2014-05-22

**Authors:** Mario López-Pérez, Ana-Belen Martin-Cuadrado, Francisco Rodriguez-Valera

**Affiliations:** Evolutionary Genomics Group, División de Microbiología, Universidad Miguel HernándezAlicante, Spain

**Keywords:** homologous recombination, SNP, genomic island, aquatic bacteria, phage predation, genomic diversity

## Abstract

Different strains of the same prokaryotic species, even very similar ones, vary in large regions of their genomes. This flexible genome represents a huge reservoir of diversity that allows prokaryotes to exploit their environment efficiently. Most of the flexible genome is concentrated in genomic islands, some of which are present in all the strains and coding for similar functions but containing different genes. These replacement genomic islands are typically involved in exposed cellular structures, and their diversity has been connected to their recognition as targets by prokaryotic viruses (phages). We have compared genomes of closely related aquatic microbes from different origins and found examples of recent replacement of some of these flexible genomic islands. In all cases, that include Gram positive and negative bacteria and one archaeon, the replaced regions boundaries contain tell-tale peaks of increased, mostly synonymous, nucleotide substitutions. They tended to be sharper at the boundary closest to the origin of replication of the island. We will present the hypothesis that replacement flexible genomic islands are often exchanged by homologous recombination between different clonal frames. These recombination events are possibly selected due to the immediate reward provided by a change in the phage sensitivity spectrum.

## Introduction

A main driver of intra-species diversity in prokaryotes is the presence in different strains of the same species of different gene complements coding for completely different functions (see for example Gordienko et al., 2013). This is a major difference with eukaryotes in which most of the within species genomic diversity derives from the presence of different alleles of the same genes. This generates a remarkable within species diversity of phenotypes with different physiological, ecological, and pathogenic properties (Hazen et al., 2013). The biological role of this broad genetic diversity is a critical issue to understand the biology of prokaryotes. Actually, that such high diversity can be found in concurrent (derived from the same sample) free living microbes (Gonzaga et al., 2012; López-Pérez et al., 2013) is puzzling unless radically novel perspectives about populations genomics of prokaryotic microbes are conceived (see for example Rodriguez-Valera and Ussery, 2012; Cordero and Polz, 2014).

Most of the differential gene complement (typically 60–80% of the flexible genes) is concentrated in genomic islands, i.e., clusters of genes of more than 10 Kb (Mira et al., 2010; Gonzaga et al., 2012; López-Pérez et al., 2013). Some of these islands tend to be found at the same genomic location and context although they are sometimes completely absent from some strains. We have named this kind of island flexible Genomic Islands (fGI) (Gonzaga et al., 2012), fGIs are genomic regions that vary from one strain to another within the same species although (1) are located at equivalent positions (2) with the same neighboring core genes and (3) often with similar inferred function. Recently, the sequencing and availability of more than one complete genome belonging to the same clonal frames (CFs) of the same species, allowed the distinction of two different types of fGIs (López-Pérez et al., 2013). One type is typically associated to mobile genetic elements (including lysogenic phages) and these genomic islands have been designated “additive,” because they typically vary among strains by the presence/absence of a different number of gene cassettes flanked by integrases or transposases, sometimes even within a specific CF. The variability found in additive fGIs is mechanistically simple, using the diverse and well-characterized mobility of these genetic elements (transposition, integration etc.) as drivers of the inter-strain diversity. Another different category are the replacement fGIs (López-Pérez et al., 2013). In them we found completely different gene clusters in the different CFs. However, they had similar assigned functions, and were at the same genomic location. They are typically involved in structural, often critical for survival, features of the cells that are exposed to the environment. The gene cluster coding for the O-chain polysaccharide (sometimes called O-antigen) of the Gram negative lipopolysaccharide (LPS) is a paradigmatic example (Nazarenko et al., 2011; Gonzaga et al., 2012; Rodriguez-Valera and Ussery, 2012). Other gene clusters that follow this type of dynamics are the flagellum glycosylation island, the exopolysaccharide (or capsular polysaccharide) or the giant protein gene clusters (Gonzaga et al., 2012; López-Pérez et al., 2012, 2013). The mechanism of diversity generation in replacement fGIs is not as clear as in the case of additive fGIs. There are no clear hall marks of illegitimate recombination at the boundaries and, although some of these fGIs contain IS elements, they are not particularly prevalent in these genomic regions (Cuadros-Orellana et al., 2007; Gonzaga et al., 2012). On the other hand, the similarity throughout the different region is often negligible and makes homologous recombination an unlikely event. Recently, we found accidentally a clear case of a recent exchange of a replacement fGI. The gene cluster codes in this case for the genes involved in the flagellum glycosylation of *Alteromonas macleodii* (López-Pérez et al., 2013). The fGIs were identical for two strains of significantly divergent genomes over their homologous regions. This could be an example of a “smoking gun,” i.e., a very recent event of replacement. In addition, the bordering areas showed a high accumulation of single nucleotide polymorphisms (SNPs), most of them synonymous. The small d*N*/d*S* ratios that were found for the bordering genes were a strong indication that the SNP increase found at these genes were due to frequent recombination events with other microbes even more divergent than the two strains considered. Recombination at both ends of the island might elicit the complete replacement of the gene cluster.

To examine how widespread was this phenomenon we have systematically searched genomes of aquatic microbes of different origins for which more than one finished and complete genome (rather than draft) was available, to detect similar examples of recent recombination affecting replacement fGIs. The results point to a model of homologous recombination akin to the crossing over of eukaryotes that uses the high similarity of the surrounding areas to precisely replace the contents of the fGI by one present in a different strain.

## Materials and methods

### Genomic data

The first step in this analysis was to search in the literature groups of aquatic microbes with some strains of the same species sequenced. Then, all the genomes available of each group were downloaded from the NCBI FTP site (http://ftp.ncbi.nih.gov/genomes/Bacteria/).

### Genome comparison

From the genbank file of each genome obtained from the NCBI, Geneious Pro 5.0.1 (with default parameters) was used to locate and extract the interesting fGIs in a separate file. Reciprocal BLASTN comparisons between the selected fGIs were carried out, leading to the identification of the similarity among them. Artemis v.12 and Artemis Comparison Tool ACTv.9 (Carver et al., 2008) were used to allow the interactive visualization of genomic fragments that showed high similarity. The average nucleotide identity (ANI) between strains was calculated using JSpecies software package v1.2.1 using default parameters (Richter and Rosselló-Móra, 2009).

### SNP analysis

The numbers of SNPs between whole genomes were identified using the web-based program SNPsFinder (Song et al., 2005). Once the interesting regions were located, nucmer program in the MUMmer3+ package (Kurtz et al., 2004) was used to identify the indels and the SNPs between small regions of the genomes. The total number of SNPs in the genome was calculated for each pair of genomes to obtain the average in a 500 bp window.

### d*N*/d*S* analysis

The ratio of synonymous and non-synonymous (d*S* and d*N*) was used to quantify selection pressures acting on protein-coding regions. A low ratio (d*N*/d*S*<1) indicates purifying selection, whereas a high ratio (d*N*/d*S*>1) is a clear signal of diversifying selection. Orthologous protein sequence pairs were aligned using ClustalW and the protein alignments imposed upon the nucleotide sequences using the program pal2nal (Suyama et al., 2006). For each sequence pair, pairwise d*N*, d*S*, and d*N*/d*S* indexes were estimated by maximum likelihood using the codeml program (Yang, 1998).

## Results

### Detection of identical clusters between different CFs

The clearest evidence that homologous recombination is taking place among different strains is the presence of identical versions of replacement fGIs in different CFs, i.e., lineages that belonging to the same species are widely divergent at the level of SNPs. The contrasting absence of SNPs in the exchanged region can be taken as an indication that it has been exchanged recently (compared to the age of divergence of the rest of the genome). As mentioned before, this type of “smoking gun” situation has been found in isolates of the marine bacterium *A. macleodii* (López-Pérez et al., 2013). Therefore, we have carried out a literature search for aquatic microbes with more than one strain of the same species fully sequenced and at least one complete assembled genome. This way, we have found a number of recent events of exchange of replacement genomic islands that are listed in Table 1. In all cases, the island was identical between two strains that, although clearly belonging to the same species (>98% ANI), were different (3–4 SNPs per 500 bp over their core genomes). However, the exchanged fGIs always maintained a nearly identical sequence containing only a few SNPs, indicating a very recent replacement. In all cases we found a marked increase in synonymous replacements taking place in the bordering shared regions next to the fGIs. The increase in SNPs was often sharper in the side closest to the origin of replication. We have referred this region as origin side (OS) and the farthest as terminus side (TS). The average d*N*/d*S* substitution rates for the neighboring genes was between 0.09 and 0.001 (Table 1), indicating strong purifying selection. As a reference the average values for this parameter along pairs of strains shared genomic regions range typically between 0.125 and 0.188 (Jordan et al., 2002; Pena et al., 2010; Gonzaga et al., 2012; Martincorena et al., 2012).

**Table 1:** **Comparison of fGIs found in different clonal frames of the same species**.

**Species**	**#genomes analyzed**	**Denomination of the strains compared**		**ANI (%)**	**#SNPs (per genome)**	#SNP (genome average)^*^	#SNPs found at the boundaries^**^				**d*N***	**d*S***	**d*N*/d*S***	**Inferred protein function of the gen having the SNPs peak**
							**Identical gene cluster at the fGI**							
							**O-chain/S-layer cluster**		**Flagellum cluster**					
							**OS**	**TS**	**OS**	**TS**				
A. macleodii^***^														
	11	615	U8	98.4	30913	3.5			93	15	0.034	1.165	0.029	FlgH
***A. veronii***														
	7	AMC35	B565	97.8	19600^****^	2.2	47	20			0.000	0.111	0.001	DNA-binding protein
		AMC35	AER397	98.1	21516^****^	2.4	47	37			0.000	0.100	0.001	DNA-binding protein
***Ca.* Pelagibacter ubique**														
	9	HTCC 1002	HTCC 1062	97.6	13762	5.2	38	74			0.002	0.481	0.005	PntB
***V. cholerae***														
	20	O395	O1	99.3	13592	2.2	29	10			0.000	0.100	0.001	Dfp
***V. vulnificus***														
	10	MO6-24/O	CMCP6	98.9	34037	5.1	89	26			0.004	0.108	0.005	lipoprotein
***S. arenicola***														
	25	CNX481	CNT857	98.2	2084^***^	3.3	56	16			–	–	–	Intergenic region
							**Different gene cluster at the fGI**							
***A. macleodii***														
	11	DE	DE1	98.6	23225	2.6	154	27			0.012	1.132	0.011	UDP-GlcA epimerase
		DE	AD1000-35-C2	–	–	–	57	–			0.003	0.302	0.010	UDP-GlcA epimerase
***P. aeruginosa***														
	14	NCGM2.S1	PAO1	98.6	53064	3.9			107	9	0.215	3.032	0.070	FlgL
		DK2	M18	99.3	25709	2			108	11	0.203	2.987	0.060	FlgL
***H. walsbyi***														
	4	HSBQ001	C23	98.7	34453	5.5	37	91			0.205	2.156		

* SNP in a 500 bp window [(#SNPs/genome length) × 500];

** Highest value of the previous parameter in the fGI

*** Data from López-Pérez et al. (2013);

**** draft genome; PntB, beta subunit of the NAD (P) transhydrogenase; Dfp, phosphopantothenoylcysteine decarboxylase; UDP-GlcA epimerase, UDP-glucuronic acid epimerase. OS, Origin side; TS, Terminus side.

**Figure 1:**
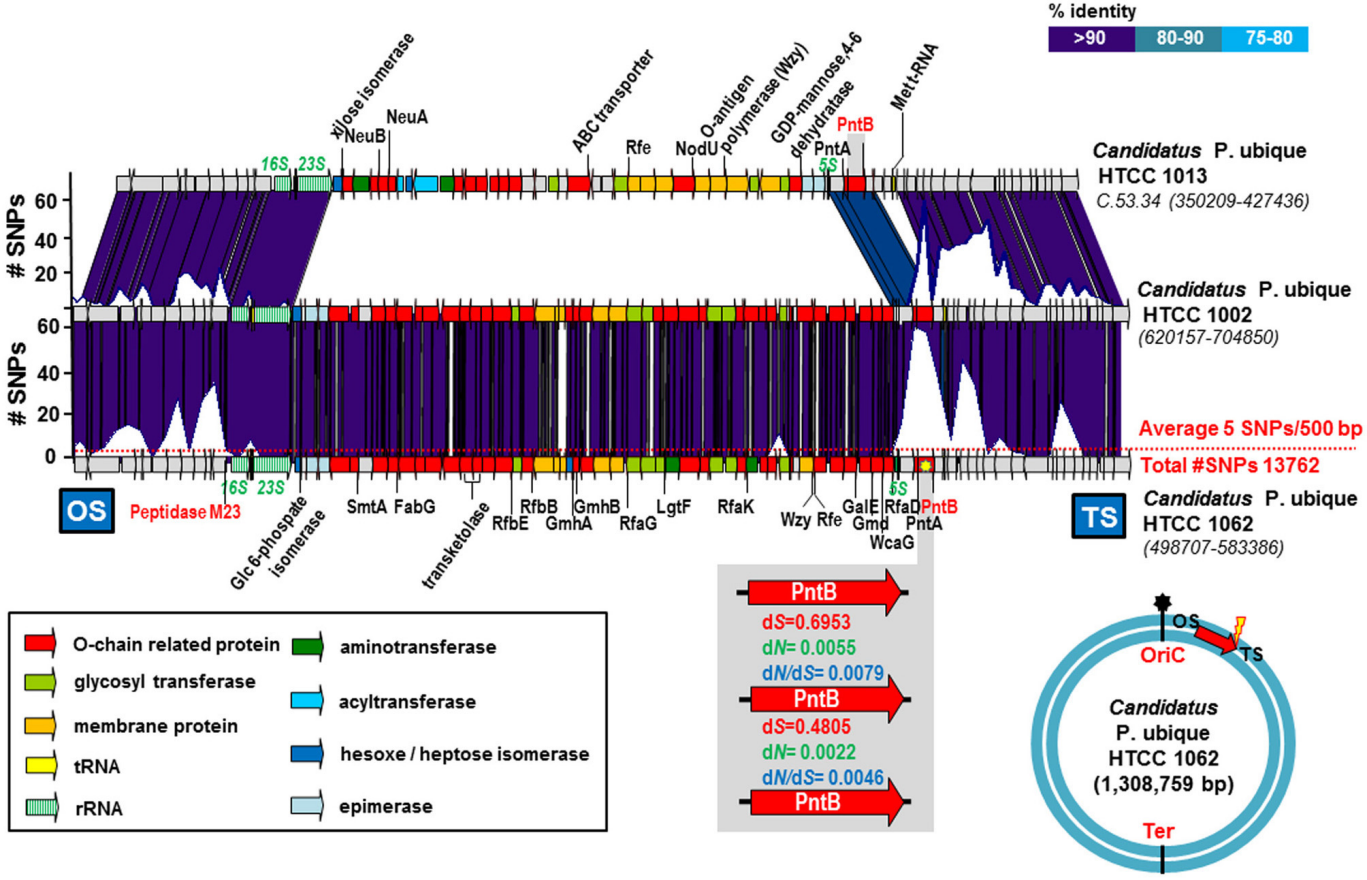
***Candidatus* Pelagibacter ubique HTCC 1013, 1002, and 1062 O-chain cluster**. The plot above the genomes indicates the number of SNPs in a 500 bp window. The average number of SNPs in the genome is indicated by a red dot line. The region enlarged in the gray box indicates the rate of non-synonymous (d*N*), synonymous (d*S*) substitutions and the d*N*/d*S* ratios for the gene which accumulates the largest number of SNPs that encodes a PntB: beta subunit of the NAD (P) transhydrogenase (marked with a yellow asterisk). The blue circle indicates the position of the gene cluster in the *Candidatus* Pelagibacter ubique HTCC1062 genome. The origin of replication (oriC) has been manually identified, while the terminus (Ter) is just located oposite (not identified). The red arrow shows the orientation in which the gene cluster is represented. Yellow lightning indicates the side of the island where the accumulation of SNPs is highest. OS, Origin Side; TS, Terminus Side.

One example of exchange of an O-chain cluster fGI was found in the marine microbe *Candidatus* Pelagibacter ubique (Figure 1). This is a well-known free-living heterotrophic marine bacterium that has one of the smallest genomes for any free living organism (*ca*. 1.3 Mb). We found nine different genome sequences available at the NCBI. The synteny among them was well conserved and allowed the alignment of the strains and the identification of fGIs (Wilhelm et al., 2007). As has been already pointed out (Wilhelm et al., 2007) the two strains HTCC 1002 and HTCC 1062 have identical versions of the O-chain gene cluster. However, other genomes (see for example that of strain HTCC 1013 in Figure 1) contained a completely different O-chain gene cluster. HTCC 1002 and HTCC 1062 both came from the same sample located at a temperate coastal Northeast Pacific site (Rappe et al., 2002). However, the ANI between them was 97.6%, indicating that both strains belong to different CFs within the same species. Interestingly, only nine SNPs were found in the O-chain cluster (52 Kb), even though the genomes differ by an average of 5.2 SNPs per 500 bp (a total of 13762 SNPs) (Figure 1). In addition, a large number of synonymous SNPs were detected at the boundaries of the island, which are well defined due to the conservation with the other sequenced strains (Figure 1). The genes found at the conserved boundary are, *pntB* that codes for the beta subunit of the NAD (P) transhydrogenase (an integral membrane protein that is involved in the synthesis of NADPH through the membrane translocation of protons) (Jackson, 2003) at the OS side, and a gene coding for a peptidase M23 at the TS. The d*N*/d*S* value indicates that the most likely reason for the high number of SNPs was recombination. Positive selection would induce much higher values of d*N/*d*S* (Castillo-Ramírez et al., 2011) (Figure 1). This fGI has been previously described and identified as one of the four hypervariable regions in the recruitment of HTCC 1062 in the metagenome sequence data from the Sargasso Sea (Wilhelm et al., 2007). It is noteworthy that the fGI included the only ribosomal operon of this microbe. It has been suggested (Wilhelm et al., 2007) that the 5S and 23S rRNA genes can act as a target of homologous recombination to provide a rapid spread of new versions of the O-chain cluster. However, we found the peak of SNPs located just before the 16S rRNA gene and after the 5S rRNA gene (Figure 1) indicating that ribosomal RNA operon was likely exchanged together with the O-chain cluster.

**Figure 2:**
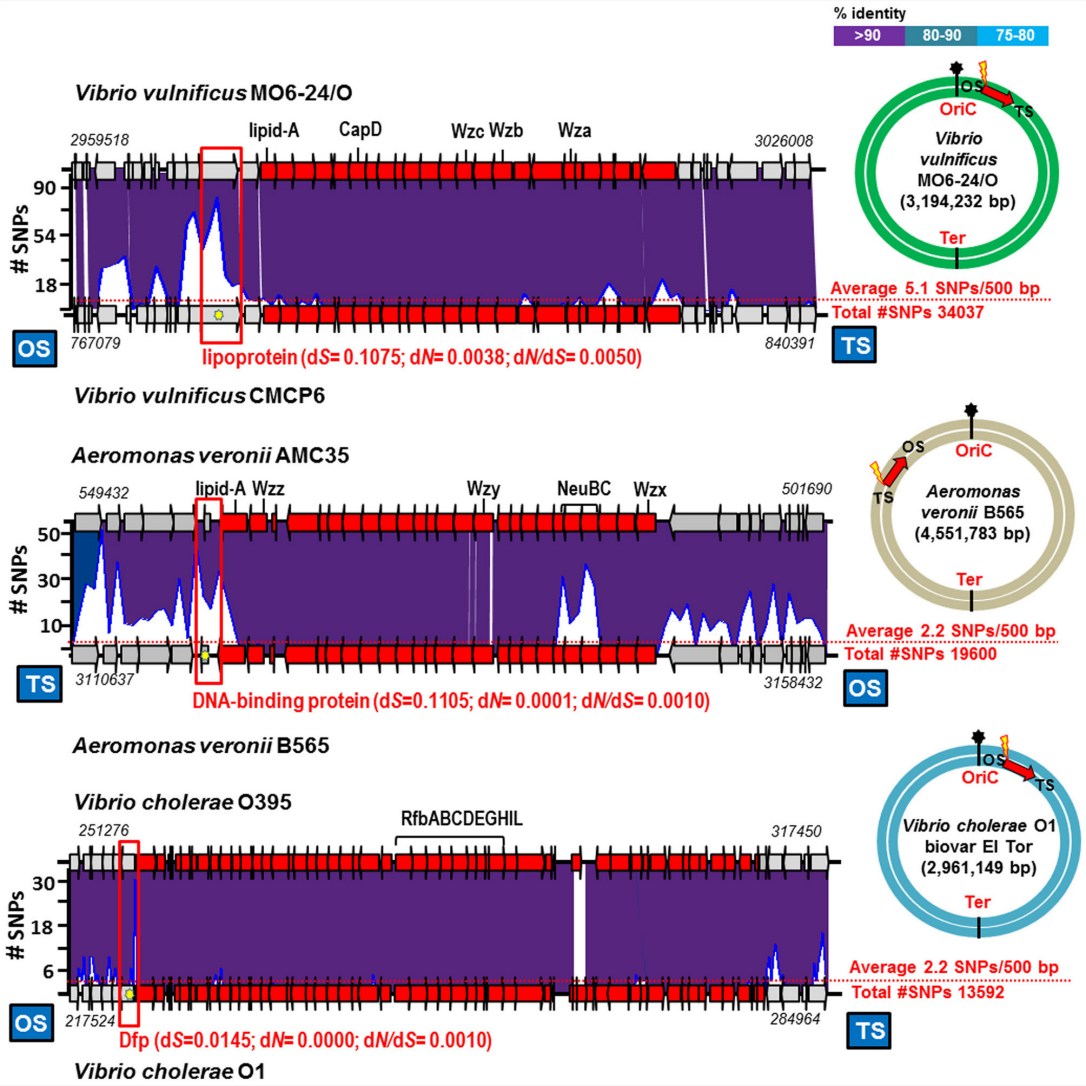
**Identical gene cluster involved in the biosynthesis of the O-chain in aquatic/pathogenic bacteria**. The plots above the lower genome indicate the number of SNPs in a 500 bp window. The average number of SNPs in the genome is indicated by a red dot line. Yellow star indicates position of the gene which has the highest number of SNPs and between brackets is shown the rate of non-synonymous (d*N*), synonymous (d*S*) substitutions and the d*N*/d*S* ratios. The colored circles show the position of the gene cluster in the genome. Location in the genome and site of maximum SNP numbers indicated like in Figure 1.

We have found similar examples of recent exchange in the comparison of *Vibrio vulnificus, Vibrio cholerae* and *Aeromonas veronii* strains, Gram negative bacteria that although sometimes are pathogenic for humans are also autochthonous to aquatic environments (Jones and Oliver, 2009; Cho et al., 2010; Janda and Abbott, 2010). We found three cases of identical O-chain clusters in pair of strains that differed by an average of 2–5 SNPs per 500 bp (Figure 2). Like in the case *of Ca*. Pelagibacter, a large accumulation of SNPs, mostly synonymous, and in these cases located just before the OS of the O-chain cluster, were detected (Table 1 and Figure 2). The O-chain gene cluster was free of SNPs, with the exception of the *A. veronii* example (AMC35 and B565), where two genes involved in the synthesis of sialic acids, located in the middle of the cluster, accumulated a few SNPs (Figure 2). These examples indicate that two major glycosylation gene clusters in Gram negative aquatic bacteria (counting also the flagellum glycosylation cluster of *A. macleodii* mentioned above) are subjected to exchange between strains that belong to the same species but differ significantly over their core genomes i.e., are different CFs.

There is prior evidence of similar situations in Gram positives. (Dingle et al., 2013) analyzed the genome of 57 *Clostridium difficile* isolates, a Gram positive, and found a high genetic diversity among the S-layer cassettes. They suggested that homologous recombination between close related strains was the underlying mechanism of this S-layer cassettes genetic exchange in order to avoid host immunity. In the same way, we searched for other examples in marine Gram positive bacteria. We analyzed the 25 genomes available for the obligate marine actinomycete *Salinispora arenicola.* We identified and compared the S-layer gene clusters of two strains *S. arenicola*, CNX481 and CNT857 (Table 1) with identical S-layer clusters over backgrounds that indicated different CFs (although being draft genomes a precise delimitation of the core could not be carried out). Again in this case, the tell-tale SNP peaks at the boundaries of the fGI were prominent. Suggesting that the same pattern observed to the LPS O-chain of Gram negative bacteria applies to the S-layer of Gram positives.

### Evidence for homologous recombination when different gene clusters are present at the fGIs

**Figure 3:**
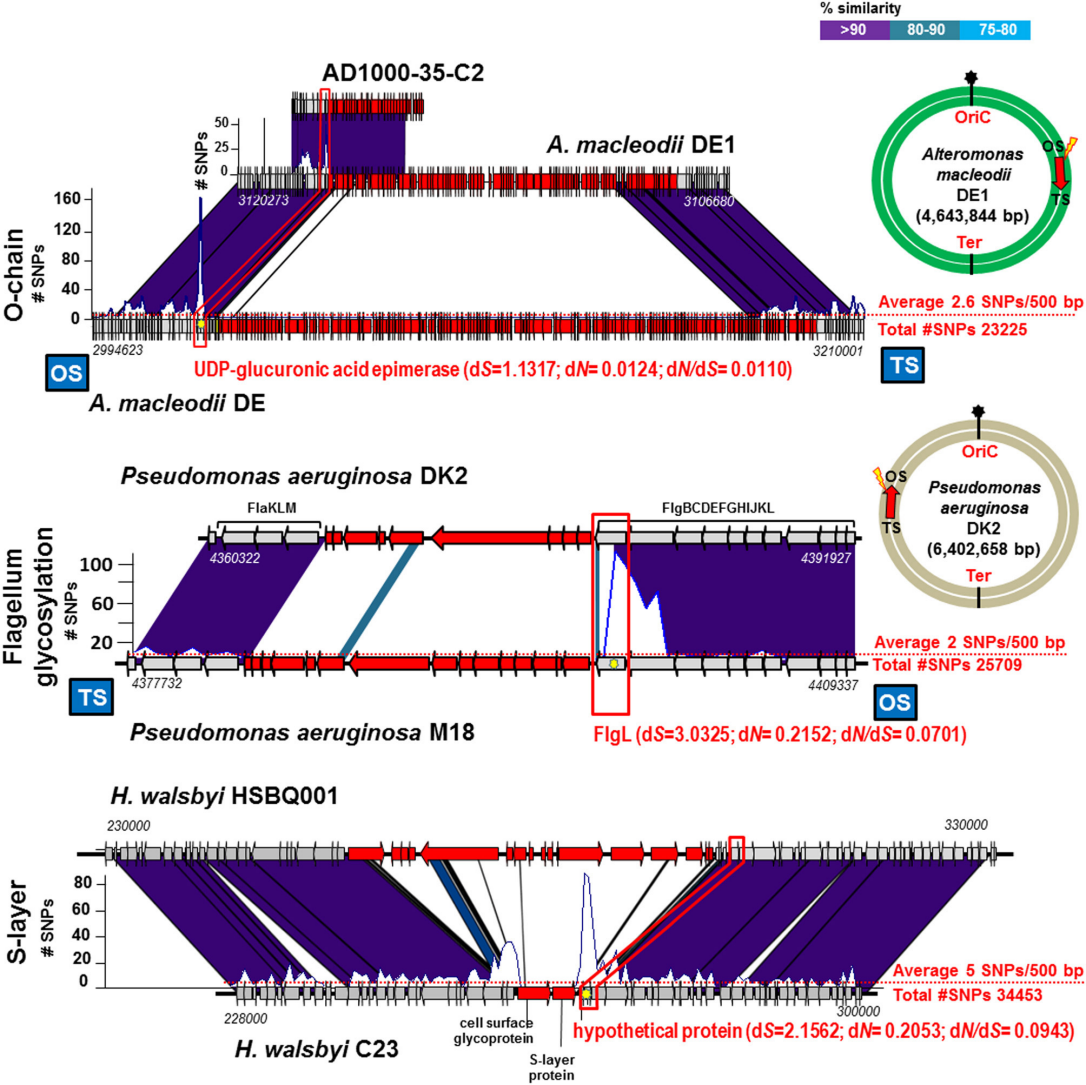
**Different versions of the same replacement fGI between pairs of strains of the same species**. Designation of the fGI compared in each example are indicated to the left. Representation of the graphic is like in Figures 1, 2.

We have examined the Gram negative groups mentioned in the previous section to find out if the sharp increase in synonymous SNPs were also apparent even when different versions of the fGI was found (as is usually the case when comparing different strains). Specifically we have checked the fGIs coding for the O-chain and flagellar glycosylation islands (Table 1 and Figure 3). Our analysis indicates that the numbers of SNPs found in the bordering areas were systematically higher. Actually, when comparing identical fGIs (see previous section) the d*S* values of the bordering genes were between 0.1 and 1.0. In the case of finding different gene clusters in the fGI, the values were typically larger than 1.0 (Table 1). In order to provide a reference average value to estimate the significance of these numbers, we analyzed the nucleotide change ratios affecting the genes of the 10 Kb syntenic regions at both sides adjacent to the fGIs (Table 2). In all cases, the peak of d*S* values observed at the boundary gene were between 4 and 34 times higher than the average for the adjacent 10 Kb. Remarkably, fGIs showed the highest density of SNPs tended to be located at the OS. The same pattern appears in most of the cases suggesting that the homologous region that starts the crossovers is typically the one located at the OS on the leading strand.

**Table 2:** **d*N* and d*S* values for the genes in the 10 Kb adjacent to both sides of the fGI**.

**Species**	**Strains compared**		**fGI**	**d*N***	**d*S***	**d*N*/d*S***
*Alteromonas macleodii*	615	U8	Flagellum	0.0064	0.0410	0.1560
	DE	DE1	Flagellum	0.0058	0.0590	0.0980
*Ca.* Pelagibacter ubique	HTCC 1002	HTCC 1062	O-chain	0.0100	0.0860	0.1160
*Vibrio cholerae*	O395	O1	O-chain	0.0016	0.0300	0.0360
*Vibrio vulnificus*	MO6-24/O	CMCP6	O-chain	0.0017	0.0300	0.0400
*Pseudomonas aeruginosa*	DK2	M18	Flagellum	0.0005	0.0979	0.0049
*Haloquadratum walsbyi*	HSBQ001	C23	S-layer	0.0097	0.1025	0.0944

fGI: flexible Genomic Island.

Archaea are a different life kingdom with overall different molecular biology. However, we have found evidence of a similar recombination event involving a surface structure gene cluster. We have analyzed the regions of a fGI detected in the halophilic archaeon *Haloquadratum walsbyi* (Bolhuis et al., 2006). This microbe is found in large numbers in saturated brines worldwide. The presence in this microbe of fGIs was detected before by comparing a strain genome with metagenomes from the same habitat and locations from which the strain was isolated (Legault et al., 2006). Notorious among them was the fGI1 that has been shown to contain the main cell surface component of this microbe, a cell surface glycoprotein gene (Dyall-Smith et al., 2011). Figure 3 shows the pattern found before for major components of the outer layer of Gram negative bacteria are reproduced here in an archaeon. The ANI between HSBQ001 and C23 strains was 98.7%, indicating that they belong to different CFs of the same species. The genomes differed by an average of 5.5 SNPs per 500 bp (34453 SNPs over the core). Located just after the TS of the S-layer cluster, a gene that codes for a hypothetical protein showed an increase of SNPs. Besides, the d*N*/d*S* was very low (0.0943), indicating mostly synonymous replacements. This data indicate that the phenomenon described here is applicable to Gram positive and Gram negative bacteria and even to Archaea.

## Discussion

The data presented here indicates that fGIs of the replacement kind, coding for surface structures and their glycosylation, appear to be frequent targets of homologous recombination. We have found evidence for similar recombination events affecting aquatic Gram negative and Gram positive bacteria and even Archaea. The regions exchanged always included the complete gene cluster required for the function rather than individual genes. Actually the exchanged region contains genes that, although are functional equivalents, i.e., code for the biosynthesis of the same structure, have very little (if any) similarity. Even the number of the genes and the sizes of these “equivalent” islands are different. The gene cluster that codes for the biosynthesis of the O-chain of the LPS in Gram negatives is the clearest example. Some genes are shared but others are completely different and actually code for the synthesis and incorporation of different sugars in the polysaccharide. In all the cases detected here, the exchange occurred between strains that, although consistently different along the genome, were closely related (typically more than 96% ANI). There is abundant evidence from the literature that in both, archaea and bacteria, recombination among related microbes at this level of similarity occurs with regularity (Vos and Didelot, 2008; Shapiro et al., 2012). What is special about the replacement fGIs is that (1) the exchanged sequence is completely different among the strains belonging to different CFs and (2) that it must happen relatively frequently as reflected by the nearly perfect sequence conservation, even at the level of synonymous mutations, between the exchanged regions. The recombination detected took place always in the conserved boundaries that are present in all the strains. Once it has been established that it was not an isolated event, this hypothesis can be re assessed as the number of complete genomes in databases increase. We found also a peculiar increase in the numbers of synonymous replacements in some shared neighboring genes that might be the target of the recombination leading to the exchange. Furthermore, this increase tended to be sharper at the OS side. In bacterial genomes the major pathway of genetic recombination are normally associated to the RecBCD enzyme (Dillingham and Kowalczykowski, 2008). The extension of this enzyme occurs co-directional with the replication loop migration to prevent collisions between the replication and recombination machinery and might be involved in this phenomenon. In Archaea the replication has multiple origins and hence no directional preference was expected, as was the case in the single archaeon studied here. In the case of *Ca.* Pelagibacter the SNPs peaks at both ends were also atypical in that they were slightly higher at the origin distal end. But this microbe is extremely slow growing and the phenomenon described above might be irrelevant.

An important issue is how frequently the recombination events described here take place in nature. Although there is a lack of precise values of the rate of change of bacteria in nature, some recent estimates in Gram negative bacteria provide figures close to 1 SNP per Mb and year (Mutreja et al., 2011; Reeves et al., 2011; Holch et al., 2013; López-Pérez et al., 2013). If we extrapolate this figure to genomic islands that are typically 20–40 Kb the time required to acquire a single SNP by the recombined fGIs would be close to a 100 years. The exchanged fGIs had very few SNPs but their number was significant enough to infer that they have not happened at shorter time frames than the 100 years mentioned above. This indicates that recombination among these islands is much too slow to be significant at the ecologically relevant range (as a phage resistance strategy) as has been argued by some authors (Cordero and Polz, 2014). In our view, these recombination events are evolutionary strategies that might have an effect in the long range i.e., after many generations and involving too much time for a short term survival strategy.

What could be the evolutionary force that drives this inter-lineage diversity and frequent exchange? We would like to speculate that this phenomenon is a reflection of the complex phage-host interaction. The clusters that have been identified as exchanged always code for exposed structures that have been identified as major phage recognition targets (Rodriguez-Valera et al., 2009; Avrani et al., 2011). The exchange of one such region possibly produces a change of the phage-sensitivity range of the bacterial lineage. This would lead to an improvement of the prey-predator equilibrium in favor of the recombinant lineage (Rodriguez-Valera et al., 2009). Of course, it can be argued that if the donor lineage was available for the exchange so will be the phages recognizing the donor receptor and the advantage would be short-lived. However, it is possible that many of the mechanisms required for a successful phage infection are cytoplasmic or simply not related to the primary receptor and thus the advantage might still be significant. In other words, the recombinant lineage would have exchanged a specialized intimately adapted phage by a newer likely more inefficient predator. A more convoluted explanation would be the recruitment of more exotic genetic material. Phages also act as gene transfer elements between cell lineages. Hence, the acquisition of a different set of infection phages might lead to the increased import of genetic material coming from a different CF, something akin to preventing inbreeding in a eukaryote. This would lead to the acquisition of a richer diversity of horizontally acquired genes and accelerate the evolutionary diversification of reproductively clonal microbes (Winstel et al., 2013).

## Author contributions

Francisco Rodriguez-Valera conceived the work. Mario López-Pérez and Ana-Belen Martin-Cuadrado performed all the analysis. Francisco Rodriguez-Valera and Mario López-Pérez wrote the manuscript.

### Conflict of interest statement

The authors declare that the research was conducted in the absence of any commercial or financial relationships that could be construed as a potential conflict of interest.

## References

[B1] Avrani S. Wurtzel O. Sharon I. Sorek R. Lindell D. (2011). Genomic island variability facilitates *Prochlorococcus*-virus coexistence. Nature 474, 604–608 10.1038/nature10172 21720364

[B2] Bolhuis H. Palm P. Wende A. Falb M. Rampp M. Rodriguez-Valera F. (2006). The genome of the square archaeon *Haloquadratum walsbyi:* life at the limits of water activity. BMC Genomics 7:169 10.1186/1471-2164-7-169 16820047 PMC1544339

[B3] Carver T. Berriman M. Tivey A. Patel C. Böhme U. Barrell B. G. (2008). Artemis and ACT: viewing, annotating and comparing sequences stored in a relational database. Bioinformatics 24, 2672–2676 10.1093/bioinformatics/btn529 18845581 PMC2606163

[B4] Castillo-Ramírez S. Harris S. R. Holden M. T. G. He M. Parkhill J. Bentley S. D. (2011). The impact of recombination on dN/dS within recently emerged bacterial clones. PLoS Pathog. 7:e1002129 10.1371/journal.ppat.1002129 21779170 PMC3136474

[B5] Cho Y.-J. Yi H. Lee J. H. Kim D. W. Chun J. (2010). Genomic evolution of *Vibrio cholerae*. Curr. Opin. Microbiol. 13, 646–651 10.1016/j.mib.2010.08.007 20851041

[B6] Cordero O. X. Polz M. F. (2014). Explaining microbial genomic diversity in light of evolutionary ecology. Nat. Rev. Microbiol. 12, 263–273 10.1038/nrmicro3218 24590245

[B7] Cuadros-Orellana S. Martin-Cuadrado A.-B. Legault B. D'Auria G. Zhaxybayeva O. Papke R. T. (2007). Genomic plasticity in prokaryotes: the case of the square haloarchaeon. ISME J. 1, 235–245 10.1038/ismej.2007.35 18043634

[B8] Dillingham M. S. Kowalczykowski S. C. (2008). RecBCD enzyme and the repair of double-stranded DNA breaks. Microbiol. Mol. Biol. Rev. 72, 642–671 10.1128/MMBR.00020-08 19052323 PMC2593567

[B9] Dingle K. E. Didelot X. Ansari M. A. Eyre D. W. Vaughan A. Griffiths D. (2013). Recombinational switching of the *Clostridium difficile* S-Layer and a novel glycosylation gene cluster revealed by large-scale whole-genome sequencing. J. Infect. Dis. 207, 675–686 10.1093/infdis/jis734 23204167 PMC3549603

[B10] Dyall-Smith M. L. Pfeiffer F. Klee K. Palm P. Gross K. Schuster S. C. (2011). *Haloquadratum walsbyi*: limited diversity in a global pond. PLoS ONE 6:e20968 10.1371/journal.pone.0020968 21701686 PMC3119063

[B11] Gonzaga A. Martin-Cuadrado A.-B. López-Pérez M. Megumi Mizuno C. García-Heredia I. Kimes N. E. (2012). Polyclonality of concurrent natural populations of *Alteromonas macleodii*. Gen. Biol. Evol. 4, 1360–1374 10.1093/gbe/evs112 23212172 PMC3542563

[B12] Gordienko E. N. Kazanov M. D. Gelfand M. S. (2013). Evolution of pan-genomes of *Escherichia coli, Shigella* spp., and *Salmonella enterica*. J. Bacteriol. 195, 2786–2792 10.1128/JB.02285-12 23585535 PMC3697250

[B13] Hazen E. L. Suryan R. M. Santora J. A. Bograd S. J. Watanuki Y. Wilson R. P. (2013). Scales and mechanisms of marine hotspot formation. Mar. Ecol. Prog. Ser. 487, 177–183 10.3354/meps10477

[B14] Holch A. Webb K. Lukjancenko O. Ussery D. Rosenthal B. M. Gram L. (2013). Genome sequencing identifies two nearly unchanged strains of persistent *Listeria monocytogene*s isolated at two different fish processing plants sampled 6 years apart. Appl. Environ. Microbiol. 79, 2944–2951 10.1128/AEM.03715-12 23435887 PMC3623136

[B15] Jackson J. B. (2003). Proton translocation by transhydrogenase. FEBS Lett. 545, 18–24 10.1016/S0014-5793(03)00388-0 12788487

[B16] Janda J. M. Abbott S. L. (2010). The genus *Aeromonas*: taxonomy, pathogenicity, and infection. Clin. Microbiol. Rev. 23, 35–73 10.1128/CMR.00039-09 20065325 PMC2806660

[B17] Jones M. K. Oliver J. D. (2009). *Vibrio vulnificus*: disease and pathogenesis. Infect. Immun. 77, 1723–1733 10.1128/IAI.01046-08 19255188 PMC2681776

[B18] Jordan I. K. Rogozin I. B. Wolf Y. I. Koonin E. V. (2002). Microevolutionary genomics of bacteria. Theor. Popul. Biol. 61, 435–447 10.1006/tpbi.2002.1588 12167363

[B19] Kurtz S. Phillippy A. Delcher A. Smoot M. Shumway M. Antonescu C. (2004). Versatile and open software for comparing large genomes. Gen. Biol. 5:R12 10.1186/gb-2004-5-2-r12 14759262 PMC395750

[B20] Legault B. Lopez-Lopez A. Alba-Casado J. Doolittle W. F. Bolhuis H. Rodriguez-Valera F. (2006). Environmental genomics of *Haloquadratum walsbyi* in a saltern crystallizer indicates a large pool of accessory genes in an otherwise coherent species. BMC Genomics 7:171 10.1186/1471-2164-7-171 16820057 PMC1560387

[B21] López-Pérez M. Gonzaga A. Martin-Cuadrado A.-B. Onyshchenko O. Ghavidel A. Ghai R. (2012). Genomes of surface isolates of *Alteromonas macleodii*: the life of a widespread marine opportunistic copiotroph. Sci. Rep. 2:696 10.1038/srep00696 23019517 PMC3458243

[B22] López-Pérez M. Gonzaga A. Rodriguez-Valera F. (2013). Genomic diversity of deep ecotype *Alteromonas macleodii* Isolates: evidence for pan-mediterranean clonal frames. Gen. Biol. Evol. 5, 1220–1232 10.1093/gbe/evt089 23729633 PMC3698932

[B23] Martincorena I. Seshasayee A. S. N. Luscombe N. M. (2012). Evidence of non-random mutation rates suggests an evolutionary risk management strategy. Nature 485, 95–98 10.1038/nature10995 22522932

[B24] Mira A. Martín-Cuadrado A. B. D'Auria G. Rodríguez-Valera F. (2010). The bacterial pan-genome:a new paradigm in microbiology. Int. Microbiol. 13, 45–57 10.2436/20.1501.01.110 20890839

[B25] Mutreja A. Kim D. W. Thomson N. R. Connor T. R. Lee J. H. Kariuki S. (2011). Evidence for several waves of global transmission in the seventh cholera pandemic. Nature 477, 462–465 10.1038/nature10392 21866102 PMC3736323

[B26] Nazarenko E. L. Crawford R. J. Ivanova E. P. (2011). The structural diversity of carbohydrate antigens of selected gram-negative marine bacteria. Mar. Drugs 9, 1914–1954 10.3390/md9101914 22073003 PMC3210612

[B27] Pena A. Teeling H. Huerta-Cepas J. Santos F. Yarza P. Brito-Echeverria J. (2010). Fine-scale evolution: genomic, phenotypic and ecological differentiation in two coexisting *Salinibacter ruber* strains. ISME J. 4, 882–895 10.1038/ismej.2010.6 20164864

[B28] Rappe M. S. Connon S. A. Vergin K. L. Giovannoni S. J. (2002). Cultivation of the ubiquitous SAR11 marine bacterioplankton clade. Nature 418, 630–633 10.1038/nature00917 12167859

[B29] Reeves P. R. Liu B. Zhou Z. Li D. Guo D. Ren Y. (2011). Rates of mutation and host transmission for an *Escherichia coli* clone over 3 years. PLoS ONE 6:e26907 10.1371/journal.pone.0026907 22046404 PMC3203180

[B30] Richter M. Rosselló-Móra R. (2009). Shifting the genomic gold standard for the prokaryotic species definition. Proc. Natl. Acad. Sci. U.S.A. 106, 19126–19131 10.1073/pnas.0906412106 19855009 PMC2776425

[B31] Rodriguez-Valera F. Martin-Cuadrado A.-B. Rodriguez-Brito B. Pasic L. Thingstad T. F. Rohwer F. (2009). Explaining microbial population genomics through phage predation. Nat. Rev. Microbiol. 7, 828–836 10.1038/nrmicro2235 19834481

[B32] Rodriguez-Valera F. Ussery D. (2012). Is the pan-genome also a pan-selectome? F1000Res. 1:16 10.3410/f1000research.1-16.v1 24358823 PMC3782348

[B33] Shapiro B. J. Friedman J. Cordero O. X. Preheim S. P. Timberlake S. C. Szabó G. (2012). Population genomics of early events in the ecological differentiation of bacteria. Science 336, 48–51 10.1126/science.1218198 22491847 PMC3337212

[B34] Song J. Xu Y. White S. Miller K. W. P. Wolinsky M. (2005). SNPsFinder—a web-based application for genome-wide discovery of single nucleotide polymorphisms in microbial genomes. Bioinformatics 21, 2083–2084 10.1093/bioinformatics/bti176 15691853

[B35] Suyama M. Torrents D. Bork P. (2006). PAL2NAL: robust conversion of protein sequence alignments into the corresponding codon alignments. Nucleic Acids Res. 34, W609–W612 10.1093/nar/gkl315 16845082 PMC1538804

[B36] Vos M. Didelot X. (2008). A comparison of homologous recombination rates in bacteria and archaea. ISME J. 3, 199–208 10.1038/ismej.2008.93 18830278

[B37] Wilhelm L. Tripp H. J. Givan S. Smith D. Giovannoni S. (2007). Natural variation in SAR11 marine bacterioplankton genomes inferred from metagenomic data. Biol. Dir. 2, 27 10.1186/1745-6150-2-27 17988398 PMC2217521

[B38] Winstel V. Liang C. Sanchez-Carballo P. Steglich M. Munar M. Bröker B. M. (2013). Wall teichoic acid structure governs horizontal gene transfer between major bacterial pathogens. Nat. Commun. 4:2345 10.1038/ncomms3345 23965785 PMC3903184

[B39] Yang Z. (1998). Likelihood ratio tests for detecting positive selection and application to primate lysozyme evolution. Mol. Biol. Evol. 15, 568–573 10.1093/oxfordjournals.molbev.a025957 9580986

